# Incontinence Cured by Chloral Hydrate

**Published:** 1871-04

**Authors:** 


INCONTINENCE CURED BY CHLORAL HYDRATE.—Dr. William Thomson reports (*Lancet*, Nov. 19, 1870) two cases of this. One was in a girl, æt. 12, who suffered for two years with incontinence at night, and frequent desire to pass her urine during day time. Fifteen grains of the chloral, given at bedtime for two nights, relieved her; and the medicine was then continued in ten-grain doses for a fortnight, at the end of which time she was cured.

The second case was a boy, æt. 13, who had suffered from nocturnal incontinence for seven years. Fifteen grains of chloral given at bedtime at once relieved him; but the medicine was continued for a fortnight, when he was quite well, and continued so when last seen, three weeks afterward.—*American Journal of Medical Sciences.*

